# PDGFRα mediated survival of myofibroblasts inhibit satellite cell proliferation during aberrant regeneration of lacerated skeletal muscle

**DOI:** 10.1038/s41598-020-79771-4

**Published:** 2021-01-08

**Authors:** Abinaya Sundari Thooyamani, Asok Mukhopadhyay

**Affiliations:** 1 Stem Cell Biology Laboratory, National Institute of Immunology, Aruna Asaf Ali Marg, New Delhi, 110067 India; 2 Present Address: Abi Nivas, Subbanapalya Extension, Bangalore, 560043 India; 3 Present Address: AA-602, Ashabari, Patuli, Kolkata, 700094 India

**Keywords:** Cell growth, Cell signalling, Muscle stem cells

## Abstract

Aberrant regeneration or fibrosis in muscle is the denouement of deregulated cellular and molecular events that alter original tissue architecture due to accumulation of excessive extracellular matrix. The severity of the insult to the skeletal muscle determines the nature of regeneration. Numerous attempts at deciphering the mechanism underlying fibrosis and the subsequent strategies of drug therapies have yielded temporary solutions. Our intent is to understand the interaction between the myofibroblasts (MFs) and the satellite cells (SCs), during skeletal muscle regeneration. We hypothesize that MFs contribute to the impairment of SCs function by exhibiting an antagonistic influence on their proliferation. A modified laceration based skeletal muscle injury model in mouse was utilized to evaluate the dynamics between the SCs and MFs during wound healing. We show that the decline in MFs’ number through inhibition of PDGFRα signaling consequently promotes proliferation of the SCs and exhibits improved skeletal muscle remodeling. We further conclude that in situ administration of PDGFRα inhibitor prior to onset of fibrosis may attenuate aberrant regeneration. This opens new possibility for the early treatment of muscle fibrosis by specific targeting of MFs rather than transplantation of SCs.

## Introduction

Muscle is one of the sturdiest tissues in the body, which holds a capacity to withstand intense pressure and force. The skeletal muscle exhibits an incredible regeneration potential. Impaired muscle regeneration following anomalies such as accidents, war, animal attacks, etc. often resulting in loss of muscle mass, leading to physical disabilities in humans. These injuries usually require prolonged rehabilitation and are often accompanied by morbidity^1^. The innate repair mechanism restores the homeostatic architecture of the damaged tissue, which would otherwise lead to locomotive dysfunction and also a condition called fibrosis^2–4^. The physiological process of wound healing and fibrosis is well documented in different organs, though the regulatory mechanisms and cellular interactions are still quite elusive. The impaired regeneration has been speculated and, in some systems, proven to be caused due to the scarcity of mobilized stem cells or the inhibition of the activated cells by micro-environmental factors. Understanding the cellular and molecular dynamics of the post traumatic physical damages that lead to this condition has been a challenge.

The current therapies are designed to enable the functional recovery of the patients via medication and rehabilitation. The amputees have the provision of prosthetic devices^5^, though the injury has been reported to remain painful for extended duration despite repair. The corollary of severe muscle injuries is the loss of muscle mass. The best therapeutic approach would be to address the cause that impedes complete regeneration and thus preventing the replacement of lost muscle tissue with fibrotic tissue. The present therapeutic methods are based on eccentric and concentric exercise models that induce injury using intense muscle workout^6,7^ and contusions^8,9^. The most popular model of muscle injury is based on cardiotoxin injury^8^. The physiological and biochemical alterations leading to muscle fibrosis is influenced by the nature of the stress and the extent of the insult. The model chosen in this investigation was the laceration based skeletal muscle injury that closely mimics the pathophysiology of severe wounds or injuries sustained under mechanical stress. Muscle lacerations disrupt the myofibers accompanied by necrosis, causing increased tissue damage or chronic injury emanating from improper healing^9–13^. Muscle regeneration involves complex and well-orchestrated molecular signaling events between the muscle stem cells, parenchymatous cells and the infiltrating immune cells in response to the wound milieu. The limited homeostatic restorative potential following severe trauma could be attributed to the deregulated or altered signals corresponding to changes in pleiotropic cytokines that modulate various cellular functions, such as proliferation, differentiation, and apoptosis or cell death.

The satellite cells (SCs) are principal postnatal muscle stem cells whose significant contribution during regeneration is irrefutable. The myofibroblasts (MFs) are key contributors in fibrosis and it is imperative to understand the interplay between the MFs and SCs. Some studies show that MFs interact with the SCs within the niche regulating their proliferation and differentiation^14,15^. The premature depletion of MFs from the injured microenvironment leads to rapid exhaustion and early differentiation of the SCs, resulting in impaired regeneration^16^. The synergy between the SCs and the MFs is least explored. Interplay between these cells could be crucial determinant in the positive or negative regulation of myogenic cell fate. The MFs function under the predominant influence of key regulatory pathways like TGF-β, FGF, PDGF and WNT^17–22^ that have been shown to have pivotal effect on cell proliferation in conjunction with their spectrum of activities in immune response during regeneration. In this investigation we have evaluated the potential of inhibiting MF specific pathways as a therapeutic target to enhance skeletal muscle regeneration by deterring fibrosis. Our study has explored the condition of severe laceration-based muscle injury, with potential to control the outcome of regeneration via MF specific target inhibition. We ascertained PDGFR pathway is vital in regulating the survival of MFs during aberrant regeneration of skeletal muscle. We propose that small molecule-based inhibition of MF survival at the crucial phase of regeneration can reduce fibrosis. The positive feedback loop of extenuating fibrosis was increased proliferation of SCs and enhanced regeneration.

## Results

### Development of a laceration-induced skeletal muscle injury model

To closely mimic the severe injuries resulting from tissue tear or disruption on physical impact, an improvised laceration muscle injury model was established that exhibited comparable tissue damage, similar pathophysiology as that observed in mechanical skeletal muscle injuries in humans. The parameters analyzed to establish the stability of the model was histopathology and the evaluation of collagen proportionate area (CPA) of the injured tissue.

The histopathology of lacerated muscle in mice has been profiled during the infiltration of the immune cells (up to day7), the regeneration phase (up to day14), and the remodelling phase (up to day21). Day 30 of the injury was considered the endpoint of the wound healing.

**Figure 1:**
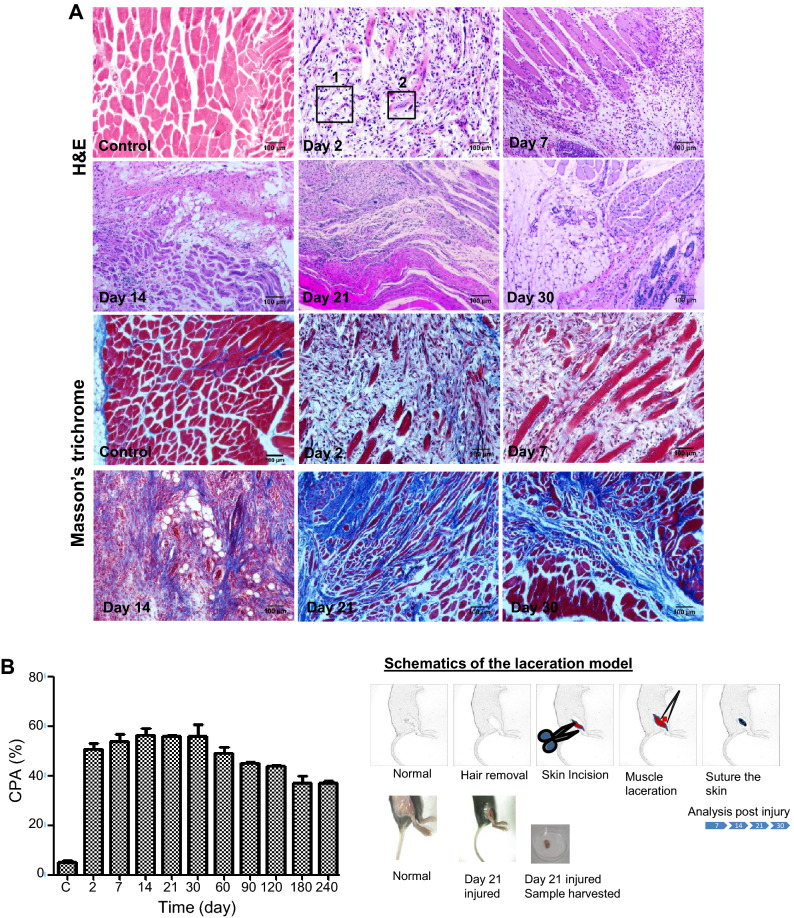
Pathology of lacerated skeletal muscle. (**A**) Histological analysis of gastrocnemius muscle post injury at different time intervals. H&E staining shows the trend of myofiber regeneration and cell infiltration. Box1—infiltrated immune cells; Box2—dedifferentiated muscle fibres (*top panel*). Masson’s trichrome staining of the sections. Results reveal heavy deposition of collagen indicated by the blue stain (*bottom panel*). (**B**) The collagen proportionate area (CPA) was quantified on the basis of morphometric analyses of 21 fields (10× magnification) of each time point using the ImageJ software. Data were presented as mean ± SEM.

The pathological observation of the injury revealed severe myofibrillar degeneration with massive infiltration of immune and other cells (Fig. 1A). The histopathology of the injured muscle has been documented for a period of 8 months to ascertain permanent fibrosis without the signs of regression (Supplementary Fig. S1A,B). The severely injured tissue revealed a varied healing pattern quite similar to that observed in Duchenne’s muscular dystrophy (DMD) in mouse^23,24^. The chronicity of the wound was further established by the continuous and elevated level of CD45^+^ cells (leukocytes) detected within the injured region, suggesting a chronic nature to the injury (Supplementary Fig. S1C). The CPA computed at specific time points of the regeneration in the fibrotic muscle revealed that 50% of the area was covered with collagen on day 2, which escalated to 60% on days 7 and 14. Day 30 exhibited collagen content ranging from 50% to 60% of the total area of the tissue (Fig. 1B). High collagen content combined with a continuous influx of immune cells confirmed the severity of the chronic injury. The fibrosis model was further validated through the gene expression analyses for well-known agonists of MFs (*Tgfβ* and *Ctgf*). The results indicated a sudden up-regulation of *Tgfβ* and *Ctgf* through the transition from day 30 to day 60. The fold-changes in gene expressions of *Tgfβ* and *Ctgf* at 60 day compared to uninjured muscle fibroblasts were 9.33 ± 0.01 and 22.49 ± 1.21, respectively (Supplementary Fig. S1D). Additionally, the presence of MFs (α-SMA^+^MFs) was observed interspersed within extracellular matrix (collagen type 1) of the injured tissue (Supplementary Fig. S2). The presence of fibrotic markers post 2 months of injury established the stability of fibrosis in laceration-induced injury model.

### Aberrant regeneration reveals a disproportionate number of MFs and SCs in the injured muscles

**Figure 2:**
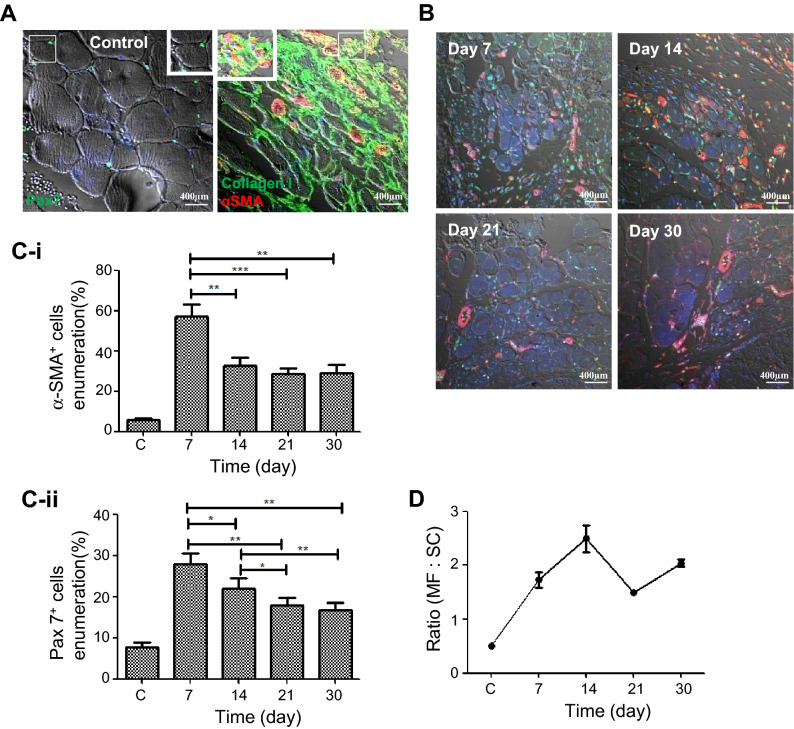
Change in MFs and SCs number within the wounded area. (**A**) Confocal images show the pathological features of the normal and fibrotic muscle. Pax7 expressing SCs are present in the sublaminar space of the normal muscle (left), whereas αSMA expressing MFs are associated with collagen type I deposits in the ECM (right) of fibrotic muscle. (**B**) Confocal images (bright field) show Pax7^+^ (green) SCs and αSMA^+^ (red) MFs at different time points of the injury. (**C-i**) and (**C-ii**) Enumeration of MFs and SCs on the basis of the expression of αSMA and Pax7, respectively, was established by morphometric analyses of 21 fields (40× magnification) using the ImageJ software. (**D**) Ratio of MFs and SCs at different stages of the injury. Data were presented as mean ± SEM. ***p < 0.001 , **p < 0.01, *p < 0.05.

To determine the pattern in the distribution of cells within the injury the numbers of SCs and MFs population were assessed. Representative images of the SCs (Pax7^+^) in the uninjured muscle, were located in the sublaminar spaces; upon injury the MFs (αSMA^+^) were densely populated in the collagen rich region of the wounded tissue (Fig. 2A). The MFs were enumeration to being the highest on day 7 of regeneration, which continued but at moderate ranges of 28.5 ± 2.42% to 32.5 ± 4.36% throughout the remaining phases of repair and remodelling (Fig. 2B,C-i). The lack of depletion of these cells and their persistence within the injury site suggests a reduced proficiency in the removal of MFs and possible defective remodelling. Concomitant enumeration of the SCs revealed a rise in their number on day 7. The SCs were accounted to 27.9 ± 2.72% of the total population, and then declined from day 14 onwards (Fig. 2B,C-ii). The number of quiescent satellite cells (Pax7^+^P57^+^) was observed to be high on day 7, which reduced over time, indicating possible depletion of quiescent cells as a result of differentiation (Supplementary Fig. S3A,B). During regeneration higher proportions of MFs to SCs were found within the wounded region. The maximum elevation was observed on day 14 of the regeneration with 2.49 ± 0.54% (Fig. 2D). The relatively higher proportion and continuous presence of MFs further substantiated the change in dynamics within the tissue resulting in aberrant regeneration. The persisting MFs and declining SC population lead us to speculate the fate of the SCs.

### Consistent increase of G_0_ state SCs during fibrosis

**Figure 3:**
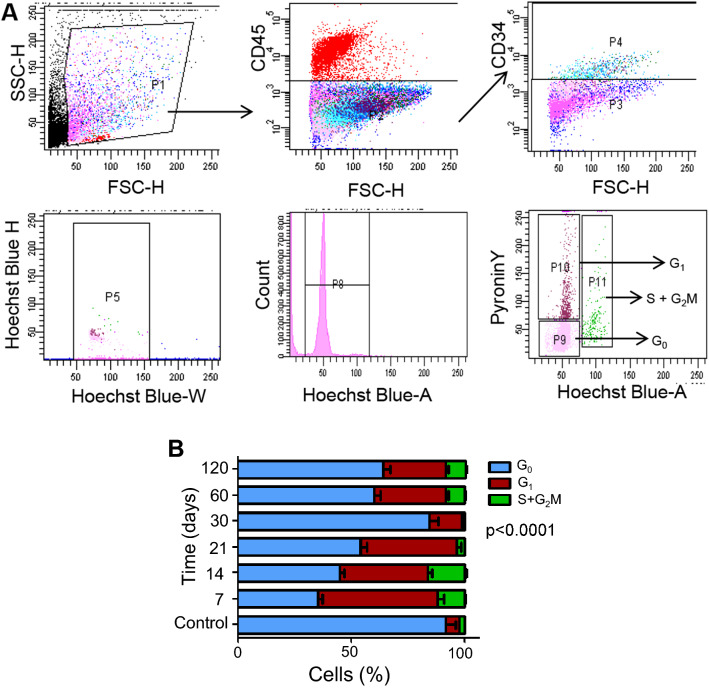
Cell cycle analysis of the SCs. (**A**) Representative dot-plots/histograms show the gating strategies followed for sorting of SCs (CD45^−^CD34^+^ phenotype) and the subsequent cell cycle analysis based on DNA and RNA quantification through Hoechst 33342 and Pyronin Y staining. (**B**) Bar diagrams represents the distinct phases of cell cycle of the SCs with respect to specific stage of injury. Data were presented as mean ± SEM.

We anticipated that the untimely exit of the SCs from cell cycle was a contributing factor to aberrant regeneration. The SCs (CD45^−^CD34^+^) were sorted and further validated by the expression of Pax7 (Supplementary Fig. S4A). The SCs were stained with Hoechst 33342 and Pyronin Y to distinguish the three cell cycling phases of G_0_, G_1_ and S + G_2_M respectively. A representative set of dot-plot is shown in Fig. 3A. Time course analyses of the cells showed progressive increase of G_0_ phase (35% to 84%), indicative of cell cycle exit (Fig. 3B). Post day 30 a deviation in the trend of G_0_ phase was noted that closely resembles the percentage observed in uninjured tissue. The cells in the corresponding cell cycle phase showed only nominal changes unlike the G_0_ phase. The activated cells observed in G_1_ phase showed fluctuation in their profile, with a significant reduction on day 30. The erratic change in trend of proliferation of SCs, with increasing number of cells exiting cell cycle across the various stages of regeneration explains the retarded wound healing. The increase in G_0_ phase cells and the subsequent reduction in G_1_ phase cells clearly indicated an absence of active proliferation of SCs that could be a contributing factor to aberrant regeneration.

### Cyclic nature of MFs proliferation within the injury indicates change in the dynamics of the microenvironment

The CD45^−^CD34^−^PDGFRα^+^ expressing MFs population was identified and further validated with αSMA expression. Most of the cells co-expressed αSMA and PDGFRα, while a few expressed either of the two markers (Supplementary Fig. S4B). Elevated number of G_0_ phase cells was observed throughout wound healing, except on day 7. A decline in G_0_ phase cells was seen post day 30 (Supplementary Fig. S4C). The parallel analysis of S + G_2_M showed reduced proliferation. Day 7 accounted 8.2 ± 0.8% CD45^−^CD34^−^PDGFRα^+^ cells in the proliferation (S + G_2_M) phase, subsequently it reduced through the phase of remodelling. Furthermore, a significant increase in proliferation was observed from day 30 (15.86 ± 0.78%) which continued till day120 (18 ± 0.52%). The number of cells in G_1_ was relatively high across the time course analysed. This showed that the MFs continued to remain active, and showed renewed proliferation post the remodelling period contributing to fibrosis. Dramatic improvement of combined fraction of activated and proliferating MFs post day 21 probably suggested the presence of recurring cues assisting their migration from adjoining tissues to the site of injury, or cues released to induce their proliferation.

### Conditioned medium of MFs 21 days post injury inhibits proliferation of SCs

**Figure 4:**
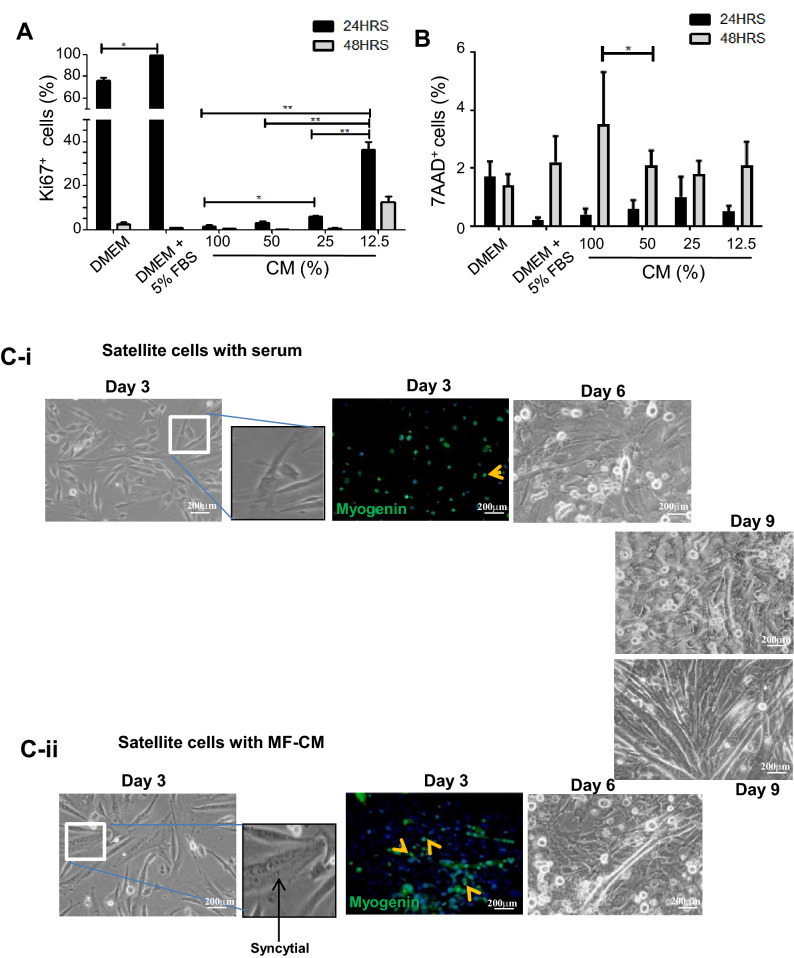
Proliferation of SCs in the presence of conditioned media of fresh and fibrotic MFs. (**A**) Proliferation of SCs in the presence of injured MFs conditioned medium (MF-CM). Isolated SCs were cultured in the presence of control medium and that supplemented with different proportions of day 21 injured muscle MF-CM for 24 and 48 h. Readout of the assay was based on Ki-67 staining of the cells. (**B**) 7AAD staining (indicative of dead cells). (**C-i**) and (**C-ii**) SCs of healthy mice were cultured in the presence of 5% serum supplemented (*top panel*) and 50% CM of freshly isolated MFs (*bottom panel*) for 9 days. Syncytial cells were stained with myogenin (indicated with yellow arrows) and documented in bright field images. Data were presented as mean ± SEM. **p < 0.01, *p < 0.05.

The main objective of this study was to determine the resultant effect of the differential cues released by the MFs during injury. Earlier experiments showed a distinct difference in MFs and SCs proliferation profile on day 21, as corroborated by histopathological and cell cycle analyses. Therefore, we hypothesized the role of MFs at this stage to be the possible influencers that alter the dynamics, contributing to fibrosis. The conditioned medium (CM) obtained from the MFs, isolated on day 21 post trauma, was utilized to culture the SCs from healthy (control) mice for 48 h. The SCs were cultured with different dilutions of MFs conditioned media (MF–CM) (12.5%, 25%, 50% and 100%) and the readouts of the experiments were proliferation and death of SCs. Increased proliferation was observed in control SC cultured in serum supplemented medium than medium devoid of serum. The proliferation radically declined in the presence of CM (Fig. 4A). Out of four dilutions examined, SCs exposed to 12.5% dilution alone showed some proliferation (36.2% Ki-67^+^ cells) in the first 24 h, after which there was a decline in proliferation (Fig. 4A). Similarly, in determining cell death, the value tended to increase with the duration of exposure to the CM, enumerated to below 1% in 24 h of culture and remained within 4% for the total duration of the analysis (48 h) (Fig. 4B). These results explained low proliferation rate as the cause of poor wound healing rather than death of SCs.

In a separate study, the effect of MFs on SCs was assessed to determine their potential to induce differentiation in the latter. It is important to mention that in these experiments both SCs and MFs were isolated from healthy mice. The control experiment was conducted in the presence of 5% serum supplemented medium, whereas in test experiments medium was supplemented with 50% MFs–CM. The cells were observed at intervals of 3 days for total of 9 days. On day 3, 80.23 ± 2.3% cells expressing myogenin, was comparable to that found in control experiments (Fig. 4C-i,ii). It may be noted that total myogenin expressing nuclei in the control culture were much lower than the test. Further analysis showed formation of numerous myotubes in test samples in comparison to the control. This provides the preliminary evidence that CM of healthy MFs contains factors that can induce differentiation of the SCs.

### Inhibition of TGFβ, NF-κB and PDGFRα signaling results in suppression of fibrosis

**Figure 5:**
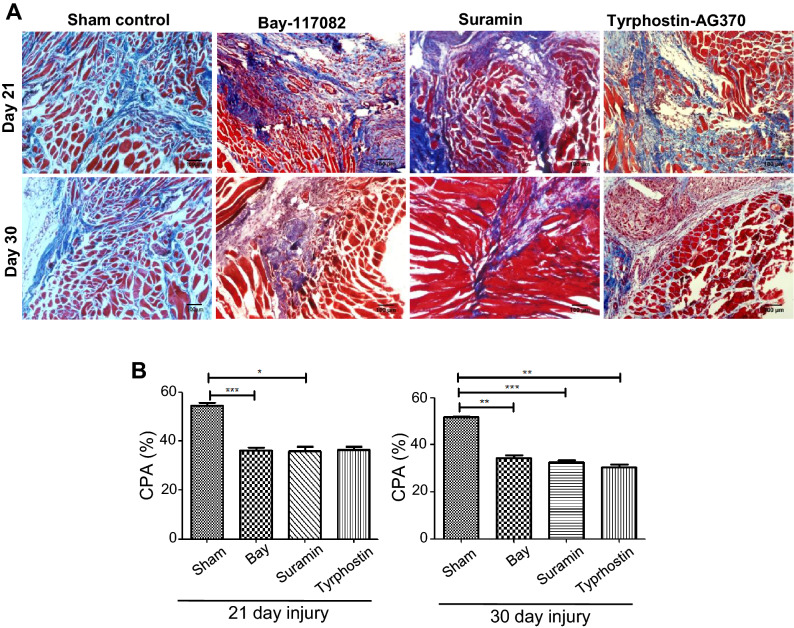
Histological evaluation of tissue sections following inhibitions of specific signaling. (**A**) Masson’s trichrome stained sections. Four groups of lacerated mice on day 14 of injury received one injection each of vehicle (sham control), Bay117082, Suramin and Tyrphostin AG-370. Seven days post injection (day 21) and 16 days (day 30) of the injury, the tissue sections were stained with Masson’s trichrome stain. (**B**) Collagen proportionate area (CPA). The CPA was calculated on the basis of morphometric analyses of 21 fields (10× magnification) of each condition using the ImageJ software. Data were presented as mean ± SEM. ***p < 0.001, **p < 0.01, *p < 0.05.

Two major signaling pathways proven to influence the course of fibrosis are TGFβ and NF-κB, of which TGFβ signaling is the most potent. PDGFRα is an upcoming contender with a potential to influence regeneration and fibrosis. We aimed at identifying the signaling pathway that might have a role in the survival of MFs and subsequently fibrosis while aiding regeneration. Post laceration on day 14 the mice were segregated into four groups, each received an on-site injection of vehicle (sham control), Bay-117082 (inhibits nuclear translocation of p65), Suramin (inhibits TGFβ receptor) and Tyrphostin-AG370 (inhibitor of PDGFRα receptor). The histopathological analyses of tissue samples obtained from drug injected groups of day 21 (7 days post inhibition) and day 30 (16 days post inhibition) of Bay117082, Suramin and Tyrphostin-AG370 inhibited groups revealed higher wound regeneration. This was observed by the newly generated fibres and the diminution of injury, when compared to the uninhibited sham control (Fig. 5A). To gain an accurate measure of the resolution of fibrosis, CPA were determined and that showed a dramatic reduction in CPA in the treated when compared to the sham control (Fig. 5B). These results suggest that treatment with any of these compounds inhibits pathological condition of fibrosis in laceration-induced muscle injury in mice.

### Inhibition of PDGFRα shows marked reduction of active MFs during the remodelling phase

**Figure 6:**
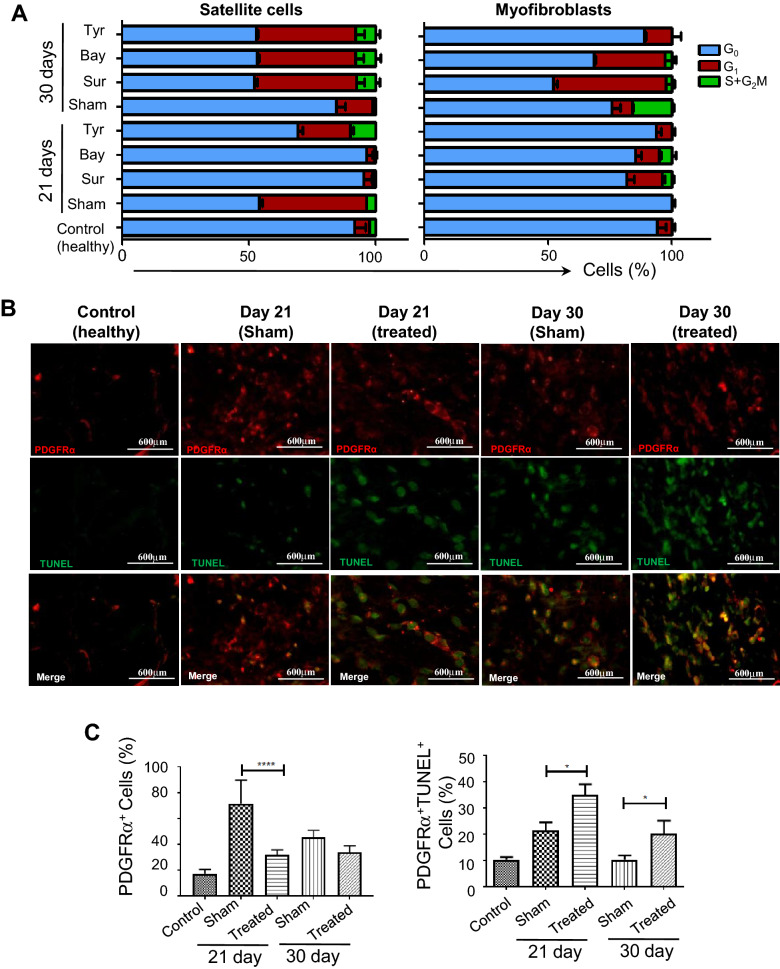
Growth inhibition and apoptosis of cells in the presence of inhibitors. (**A**) Cell cycle analysis. Experiments were conducted as above; both SCs and MFs fraction of muscle cells were analyzed for different phases of the cell cycle. Tissues were collected on days 21 and 30 post treatment with inhibitors (post days 7 and 16 of injection of drug) and without inhibitors (sham control). Separate cell cycle profiles of SCs and MFs are shown. (**B**) Apoptosis of MFs in the presence of Tyrphostin AG-370. Tissue samples (sham control and treated) were subjected to TUNEL assay post 21 and 30 days of injury (representative images—60× magnification). Bar diagrams represents the quantitative change of MFs and TUNEL-positive MFs, determined by morphometric analyses of 10 fields (20× magnification) using the ImageJ software. Data were presented as mean ± SEM. ***p < 0.001, **p < 0.01, *p < 0.05.

Since PDGFRα is potentially active in the MFs and also proven to be absent in the SCs^25,26^, it encourages further analysis of the inhibition of this receptor along with that of TGFβ and NF-κB pathways. We asked the question, whether this inhibition has any specific effect on the proliferation of either SCs or MFs. The cell cycle analyses suggest that in the case of Tyrphostin-AG370 based inhibition, the SCs showed both activation and proliferation phases on day 21, about 20.8% and 10% cells were in G_1_ and S + G_2_M phases, respectively (Fig. 6A). Interestingly, in cases of Suramin and Bay117082 based inhibition there was no proliferation of the SCs. On day 30, the cell cycle of SCs in the presence of all three inhibitors was comparable (Fig. 6A). On the other hand, no proliferating phase was detected in MFs at day 21 and 30 in the presence of Tyrphostin-AG370, which was indistinct in cases of the other two inhibitors (Fig. 6A). Above results were supported by histopathological observation, which exhibited reduction in collagen content within the wounded area (Fig. 5A). Overall, these results suggest that MFs growth was inhibited without appreciable effect on the SCs by treatment with PDGFRα inhibitor, Tyrphostin-AG370 making it a potential candidate for inhibiting fibrosis.

We predicted Tyrphostin-AG370 treated MFs undergo increased apoptosis. This was validated on tissue sections of PDGFRα inhibited group using TUNEL assay. The day 21 and day 30 tissue section results showed numerous PDGFRα^+^ cells were also TUNEL-positive within the injured region. In a comparative analysis, Tyrphostin-AG370-treated mice had much higher TUNEL and PDGFRα expressing MFs than the untreated and sham control samples (Fig. 6B). Hence, we conclude that the PDGFRα signaling inhibition in MF cells induced apoptosis. The PDGFRα expressing cells alone were enumerated in the untreated and treated chronic injured mice. The treated mice showed a considerable reduction in PDGFRα expressing cells when compared to their counterpart (Fig. 6C, bottom left). This proposes that PDGFRα pathway is the most suitable pathway to target MFs, specifically to inhibit their growth and induce apoptosis.

### Cellular apoptosis in Tyrphostin-AG370-treated MFs is potentially mediated through NF-κB and AKT pathways

**Figure 7:**
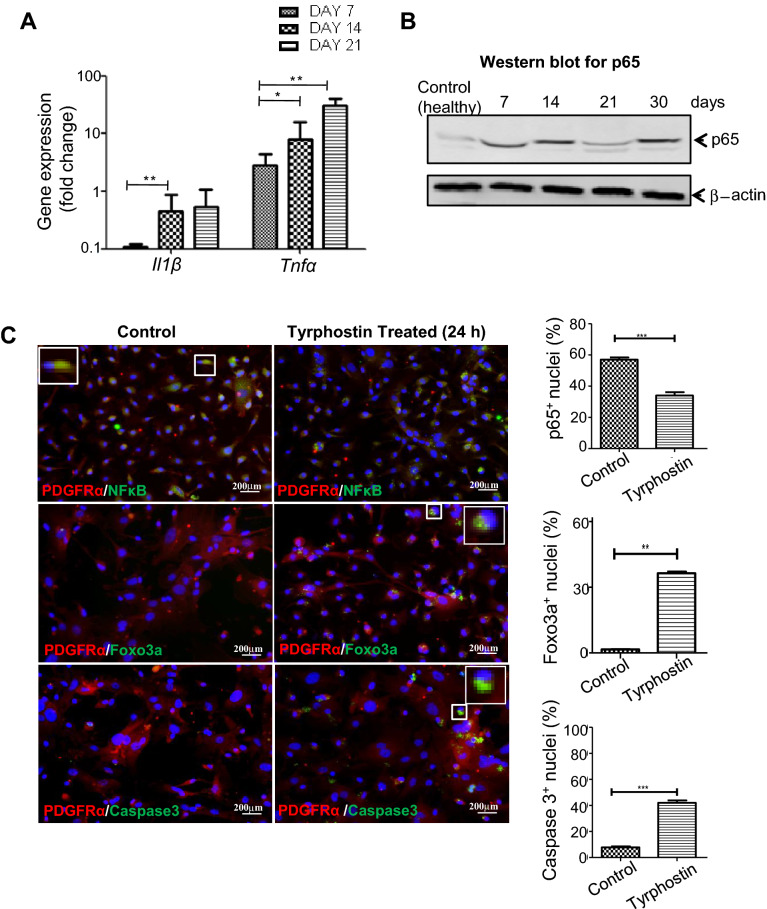
Local cues and activation of transcription factors. (**A**) Gene expression analysis. Quantitative gene expression analysis of the injured tissues shows relative mRNA of *Il-1β* and *Tnfα* expressions at days 7, 14 and 21 post muscle injury. (**B**) NF-κB activation in MFs of injured muscle. Nuclear translocation of p65 subunit at different times of the injury is shown. (**C**) Effect of Tyrphostin AG-370 on MFs in vitro. MFs are isolated from day 21 injured muscles and cultured for 24 h in the presence of plain medium (sham control) and Tyrphostin (20 μM). The resultant cells were analysed for the localization of p65NF-κB, Foxo3a and Caspase3 using specific antibodies. The bar diagrams show respective responses, determined on the basis of morphometric analyses of 6 fields each (20× magnification) using the ImageJ software. Data were presented as mean ± SEM. ***p < 0.001, **p < 0.01.

It is known that PDGFRα pathway interacts with NF-κB pathway mediating fibrosis. To decipher the mechanism, we explored the downstream effectors of PDGFRα pathway, primarily focusing on NF-κB pathway associated interactions. The well-studied canonical pathway for the activation of NF-κB is mediated by many ligands; TNFα and IL1β are most potent among them. To understand whether these ligands are over-expressed in the MFs of wounded tissue, quantitative gene expressions was carried out. The expressions of both genes were well augmented in the MFs over the course of regeneration. The expression of *Tnfα* was much higher than *Il1β* gene (Fig. 7A). In order to find out the activation of canonical NF-κB signaling, we determined nuclear localization of Phospho-p65NF-κB in MFs. It was revealed that in the MFs, NF-κB was active (Fig. 7B).

It is known that PDGFRα signaling cross-talk with both NF-κB and AKT kinases leading to disturbance in the balance of survival and apoptosis by promoting pro-survival transcription factor NF-κB and inhibiting the Foxo3a pro-apoptotic transcription factor^27^. We hypothesized that this imbalance between survival and pro-apoptotic transcripts encouraged growth and proliferation of MFs. To validate the signaling cascade, we isolated MFs from wounded tissue at 21 days and cultured it for 24 h. The cultured MFs were treated with carrier (PBS) as control or Tyrphostin- AG370 as test. The results showed that in control sample p65 was active in many cells (total cells counted: 1976–2014 nuclei) whereas Foxo3a did not localize in the nuclei (Fig. 7C). Interestingly, as expected, the cells from the Tyrphostin- AG370 treated MFs portrayed significant inactivation of p65 and activation of Foxo3a (total cells counted: 1358 nuclei), indicating the increase in pro apoptotic signals. As a consequence of that caspase-3 was active in many cells (total cells counted: 1028 nuclei) (Fig. 7C, lower panel). This explains the TUNEL assay results of MFs in the Tyrphostin- AG370 treated wounded tissue. Overall, these preliminary results described a potential mechanism by which aberrant regeneration was attenuated in the chronically injured muscle on treatment with the small molecule drug Tyrphostin-AG370.

## Discussion

The restoration of the injured tissue is the desired endpoint of any regeneration, which is often lacking when the wound is of a severe nature. The inadequacy in myofiber regeneration and the persistence of MFs are some of the established causes for aberrant regeneration^23,28^. The current therapeutic strategies are largely focused on amelioration of fibrosis as a condition^28,29^. The cellular interactions are very dynamic and hold the ability to influence the outcome of regeneration. Our study was conducted to decipher the synergy between the MFs and the SCs during aberrant regeneration, and to observe if any modifications in this interaction could lead to prevention of fibrosis while encouraging the possible reconstitution of the damaged muscle during the process of regeneration. To accomplish this, it is vital to understand the interactions between these two cells within the wound. The limitation in the readouts of most of the studies in animal models is that they do not completely mimic the severe mechanical traumas experienced by humans. The finite information available on the fate of the SCs and MFs during regeneration is also another reason for limited success in treatment for chronic muscle injury. The SCs are triggered to proliferate under various circumstances. After proliferative phase they undergo transition with visible phenotypic and genotypic changes. The modulation depends on the coordinated activation of the entire battery of muscle specific transcription factors leading to myoblast differentiation. The system that controls these changes is complex, and the attachment of these cells to extracellular matrix is essential to accomplish differentiation, and early or delayed differentiation can lead to improper regeneration^30–33^.

The survival of the MFs is attributed to the consistent influx of inflammatory cells that secrete factors enabling these cells to proliferate, thus aiding in the initiation and perpetuation of fibrosis^34–36^. The variability in markers expressed by the fibroblasts and MFs that appear in conjunction with other infiltrating cells has also presented a challenge in cellular identification^32,37,38^. In this study detailed analysis of major cell types in muscle regeneration indicated an active participation of the SCs, previously presumed to be irresponsive^39,40^. We showed through cell cycle analysis that the SCs were active throughout regeneration. The peculiarity observed was the early exit of numerous SCs during remodelling as identified by the presence of Pax7^+^p57^+^ expression and G_0_ phase cell cycle analysis, despite the continued inflammatory response (presence of CD45^+^), thus indicating the presence of antagonistic stimuli that deterred their proliferation. Further analysis of the SCs, when exposed to injured MF-CM showed that this medium not only reduced proliferation but also accelerated the myogenic differentiation of the SCs. This could explain the imbalanced ratio of MFs to SCs enumerated during the end of regeneration showing incomplete homeostasis.

The most preferred method of treatment for fibrosis is the inhibition of pathways that promote fibrosis. The prolonged activation of a few well mapped signaling pathways like TGF-β^19^, Shh^41^, Wnt^39^ and recently PDGF^42–44^ proved detrimental to tissue regeneration while promoting fibrosis. TGFβ signaling regulates cell growth, differentiation, cell death as well as apoptosis^12^; it influences *Ctgf* expression and together they influence ECM synthesis by fibroblasts^13^. There have been anti-TGFβ therapies employed to treat fibrosis in animal models which did not promote the replacement of muscle mass^45–48^. The relevance of PDGF signaling blockade has been shown to reduce renal fibrosis and more specifically PDGFRβ blockade aided by Imatinib mesylate treatment revealed a startling improvement in renal fibrosis^49^. Our data of PDGFRα blockage revealed specific targeting in the MFs leading to apoptosis. The PDGFRα mediated MFs survival mechanism functions via NF-κB signaling^20,50,51^. NF-κB was also a possible target for the pathway that could cause the dramatic shift between regeneration and fibrosis. There are several report suggesting that NF-κB performs a paradoxical role in myogenesis albeit vital in fate determination and muscle patterning^52^. This eliminated NF-κB pathway as a candidate for MF specific inhibition study. The potential of PDGFRα inhibition leading to specific targeting of the MFs followed by resolution of fibrosis was analysed using a small molecule tyrosine kinase inhibitor (Tyrphostin-AG370). There are clinical studies underway to assess the efficacy of various tyrosine kinase inhibitors though the specific mechanism targeted in the MFs present during fibrosis was unknown. We propose that PDGFR-mediated NF-κB signaling aided survival of these cells^51^. This also shows that NF-κB pathway is vital and there is more we have yet to understand regarding its various roles in regeneration. There was a significant reduction in PDGFRα-expressing cells and deactivation of NF-κB in the Tyrphostin-AG370 treatment followed by the activation of CDK inhibitor p27 and cell cycle arrest in fibroblasts^52^. Subsequently, activation of pro-apoptotic cues and further confirmation by TUNEL results verified that the cells underwent apoptosis. When the effect of the inhibitor was observed in its natural habitat (in vivo) in the presence of fibrotic conditions, it revealed a significant decrease in MFs number. The histopathological analysis indicated reduction in scar tissue formation. In addition, a positive feedback of this treatment was the increase in the number of proliferating SCs, suggesting that the SCs were not differentiating but proliferating to contribute to new myofiber formation. The reason to find a MF specific pathway inhibition would be to facilitate the treatment of degeneration by allowing the SCs to proliferate while selectively inhibiting the MFs. This would not just curtail fibrosis or possibly resolve the established fibrosis but would also enable complete regeneration.

To summarise, this work provides a model to mimic the chronic injury incurred in humans due to physical impact. The model shows a unique combination of chronic and acute response with a detailed dynamic of MFs numerically overpowering the SCs to an early cell cycle exit. On blockade of PDGFRα, the apoptosis of MFs is observed with a conjoint induction of proliferation of SCs. This leads to the resolution of fibrosis and promotion of regeneration through the specific targeting of the MFs.

## Materials and methods

### Animals

C57BL6/J mice (20–25 g) were obtained from Jackson Laboratories and maintained in the institute’s experimental animal facility. For each experiment, 12 weeks old mice were used. The plan of experiments and the number of mice used in each experiment has been mentioned in Table 1. The molecular analysis was performed on pooled mice tissue samples; the histopathological samples were obtained from individual mice. All the experiments were performed in triplicate. Animals were housed and maintained in accordance with the guidelines approved by the Institutional Animal Ethics Committee at the National Institute of Immunology, New Delhi, India.

**Table 1:**
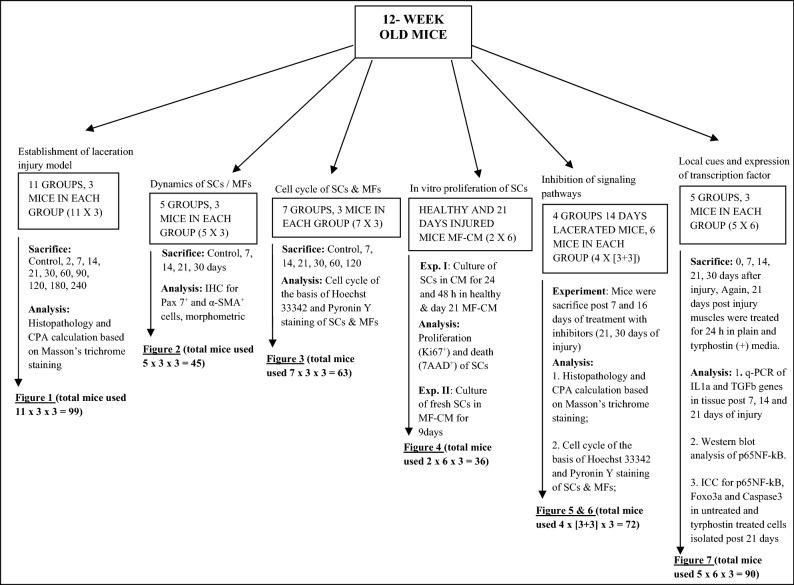
Details of plan of the experiments and mice utilized for this investigation.

### Surgical induction of mechanical injury

Mice were anesthetized with Ketamine (100 mg/kg b.w) and Xylazine (15 mg/kg b.w). The Ketamine/Xylazine cocktail was injected once 15 min prior to the surgery via the intraperitoneal route. An incision was made on the right lower limb of the mouse just alongside the anatomical position of the gastrocnemius muscle using a surgical blade. The lacerated wound size of approximately 4 mm × 4 mm × 3 mm (breadth × length × depth) was created in the gastrocnemius to induce chronic injury. The injured site was further lacerated to prolong necrosis to stabilize a chronic injury model. Briefly, we depilated the right limb at the anatomical position of the gastrocnemius muscle, an incision was made and the tissue with the above-mentioned dimensions was excised. The wound was sutured and the mouse was administered a single dose of analgesic and antibiotic via the intramuscular route: Ketoprofen (5 mg/kg b.w) and Endofloxicin (500 mg/kg b.w). The wound was covered with the antiseptic cream betadine and allowed to heal. Mice were euthanized using excess carbon dioxide, followed by cervical dislocation at the desired time-points. The mechanically injured tissue was dissected on days 2, 7, 14, 21, 30, 60, 90 120, 180, 240. The muscle on the intact collateral limb was used as control that remained uninjured. The collected tissue was fixed in 10% zinc fixative. The tissues were then embedded in paraffin block and sectioned for histological analysis.

### Histology of tissue section

Deparaffinised tissue sections (5 μm) of the injured skeletal muscle were stained with Haematoxylin & Eosin (H&E) for pathological analysis. For Masson’s trichrome staining, the tissue sections were processed in accordance to the protocol of Accustain Masson’s Trichrome Stain (Sigma, St. Louis). Briefly, the tissue sections were fixed overnight with Bouin’s fixative, following which the sections were washed under running tap water. The sections were then stained with Mayer’s Haematoxylin for 30 min, followed by wash with running tap water for 10 min. The sections were incubated with Biebrich scarlet acid fucshin solution for 5 min. Following a wash for 5 min with deionised water, the slides were treated with a mixture of phosphotungstic and phosphomolybdic acid along with deionised water (1:1:2), for a duration of 5 min. This treatment was followed by aniline blue staining for 5 min to detect the collagen content. Excessive aniline blue stain was removed by treating with a few drops of 1% acetic acid and then dipped in water to neutralize the acid. The washed sections were dehydrated with 100% isopropanol for 5 min, xylene for 5 min and then mounted using DPX. The images were captured using Olympus light microscope and the images were analysed using Image pro (Eugene, OR, USA) and Image J software. The morphometric analysis was performed on 21 fields taken at 10× magnification, in triplicate for each time point. The CPA was calculated by measuring the entire injured area. The percentage of recovery was measured for the presence of collagen (indicated by blue stain) and the muscle fibres (indicated in red), the higher the collagen deposit the lower is the recovery and vice versa, thus indicating recovery in tissue architecture.

### Isolation of skeletal muscle cells

Primary satellite cells (SCs) and myofibroblasts (MFs) were isolated from intact tibialis anterior and gastrocnemius muscle of 3 month-old wild-type (C57BL/6) mice (pooled cells of 6 mice) through magnetic activated cell sorting (MACS) technique. The cells isolated were characterized by immunofluorescence using monoclonal anti-Pax-7 and αSMA antibody. The dissected muscle tissue was subjected to 0.2% collagenase type IV (Sigma) digestion for 1 h at 37 °C in water bath. Post digestion, the suspension was double filtered through a 100 μm filter followed by a 40 μm (BD Biosciences, San Jose, CA). The cell suspension was pelleted down at 1800 rpm at 4 °C, the supernatant was discarded. The pellet was resuspended in DMEM (GIBCO) supplemented with 20% horse serum (GIBCO) and incubated at 37 °C for 1 h for recovery. The SCs were further purified by MACS system. The SCs were cultured on 1% matrigel (Corning) coated plates, in the presence of Hams-F10 (HIMEDIA, India) supplemented with 20% FBS under normoxic conditions. The MFs were cultured on 1% gelatine (Sigma) coated plates and maintained in DMEM (high glucose) supplemented with 10% serum.

### CD45 negative cell fraction and CD34 positive/negative fraction sorted by MACS

CD45 negative (CD45^−^) cells were sorted from the muscle cells of the uninjured/injured mice at different days post injury. A double-step magnetic-activated cell sorting technique using MACS LS column (Miltenyi Biotec) was followed. CD45^−^ cells fraction was obtained and re-sorted further for two separate cells fraction, the CD34^+^ and CD34^−^ fractions. The cells obtained were utilized for multiple purposes, such as co-culture experiments and gene or protein expression analyses.

### In-vitro satellite cells survival assay

The CM was obtained from MFs isolated from uninjured and injured muscle of day 21 post injury, seeded in 24-well plates. The MFs were expanded and purified as: the non muscle population. Post sort the fibroblasts were further purified from the other cells by differential plating technique. Differential plating technique: The sorted CD45^−^CD34^−^ cells were seeded in gelatine coated 100 mm culture dishes and incubated for 2 h at 37 °C cell incubator^53,54^. The non adherent cells (other cells) were discarded and the adherent cells (fibroblasts) were replaced with fresh DMEM and cultured for 12 h. The CM was harvested from the MFs after 12 h. SCs (0.1 × 10^6^) were cultured with 2 ml of medium with varying percentage of day 21 CM, obtained by serially diluting the MFs-CM with DMEM (12.5%, 25%, 50%, and 100%). In control experiments, the cells were cultured with DMEM. The SCs were cultured for 24 h and 48 h post which the cells were analyzed for cell death and proliferation by staining with 7AAD dye and Ki67 antibodies, respectively.

In a separate study, the effect of healthy MFs on SCs was assessed to determine their potential to induce differentiation in the latter. The cells growth was monitored for a period of 9 days. Here the SCs were cultured in 50% healthy MFs-CM and the control SCs were cultured with 5% serum containing medium. The cells were analysed for SCs differentiation and myotube formation, once every 3 days.

### Flow-cytometry

Single cell suspension of primary SCs and MFs (1 × 10^6^ cells/500 μl of 1% FCS containing DMEM) were stained by incubating with primary antibodies, directly conjugated with fluorochrome at 4 °C for 30 min followed by washing with PBS-BSA solution. For biotinylated and purified primary antibodies, a total of 1 h incubation at 4 °C, followed by 30 min incubation with fluorochrome labelled streptavidin and/or fluorochrome labelled secondary antibody, respectively. Cells were washed with PBS-BSA solution and analysed by flow-cytometry (FACS Aria III, BD Biosciences, San Jose, CA). The antibodies used are shown in Supplementary Table S1.

### Cell cycle analysis

Cell cycle analysis was performed by staining cells with Hoechst 33342 and Pyronin Y (Sigma) dye^53^. Staining was performed by incubating cells (1 × 10^6^ cells/500 μl of 1% FCS containing DMEM) with 5 μl Ho33342 dye (Ho—10 mg/ml) at 37 °C for 1 h. Cells were washed, re-suspended in the above medium and further stained with Pyronin Y (PY—1.6 μg/ml) by incubating at 37 °C for 1 h. Ho-PY stained cells were washed twice and stained with the antibodies (CD45-biotinylated and purified PDGFRα) for 30 min on ice. This was followed by staining with Alexafluor 700 conjugated CD34 antibody and the secondary antibodies streptavidin APCCy7 and Alexafluor 647. The cells were further incubated on ice for another 30 min prior to washing with PBS-BSA solution. Washed cells were analyzed with FACS Aria III (BD Biosciences, San Jose, CA) using customised specific band-pass filters.

### In vivo drug inhibition assay

Two weeks (day 14) post surgery mice were divided into four groups, each group was administered with a single dose of specific drug targeting TGFβ (Suramin—2.5 mg/kg b.w), NF-κB (Bay117082—2.5 mg/kg b.w) and PDGFRα (Tyrphostin AG370—2.5 mg/kg b.w) pathway, and the fourth group was the sham control. The sham control was also injected with 0.9% saline on the right limb. The drugs were diluted from the stock solution in 0.9% saline. 20 μl of the drug was injected intramuscularly with the help of micro-syringes. The animals were sacrificed to evaluate the repair and regeneration. The muscle tissue encompassing the injury was resected 7 days (day 21) and 16 days (day 30) post drug administration for further analysis.

### Immunohistochemistry

The deparaffinised tissue sections were treated with tri-sodium citrate buffer (pH—6) for 10 min in the microwave for antigen retrieval, then washed with PBS and permeabilized with 1% Triton X-100 solution in 1% PBS-BSA solution. Subsequently, the sections were washed with PBS-T followed by blocking with 3% PBS-BSA solution and further incubated with primary antibody overnight at 4 °C. The sections were washed in PBS-T followed by incubation with secondary antibody for 2 h in the dark at 4 °C. The stained and washed tissue sections were counter stained with DAPI for 10 min at room temperature, then mounted with ProLongR anti-fade reagents, covered with cover slips and observed under a Nikon Confocal laser-scanning microscope using a Plan-Apochromat 63×/1.4 oil objective. The NIS-Elements software along with IMAGE J was used for analysis. The morphometric analysis was performed on the digital images taken. In each case the total number of cells was calculated based on DAPI-positive nuclei, followed by the percentage of positive population with respective markers.

### Supplementary methods

Other methods including preparation of paraffin block, terminal deoxynucleotidyl transferase mediated nick-end labeling (TUNEL) assay, real time PCR and western blot analysis are described in the Supplementary Methods.

### Statistical analysis

Results of multiple experiments were reported as the mean ± SEM (Standard Error Mean).

The Student t test was carried out to calculate the significance between the means of two groups, and P < 0.05 was considered to find out the significance. One-way ANOVA was utilized with Tukey posthoc for multiple group analysis. All analyses were carried out using Graph-Pad Prism software.

## Supplementary Information

Supplementary Information.
