# Supplementary material for: PDGFRα mediated survival of myofibroblasts inhibit satellite cell proliferation during aberrant regeneration of lacerated skeletal muscle
Source: Sci Rep. 2021 Jan 8;11:63. doi: 10.1038/s41598-020-79771-4 (PMC7794387; doi:10.1038/s41598-020-79771-4)
Supplement: Supplementary file 1 — Supplementary Information. [file 41598_2020_79771_MOESM1_ESM.pdf]

PDGFRα mediated survival of myofibroblasts inhibit satellite cell proliferation during aberrant regeneration of lacerated skeletal muscle

by Abinaya Sundari Thooyamani and Asok Mukhopadhyay

Supplementary Fig.1 (S1)

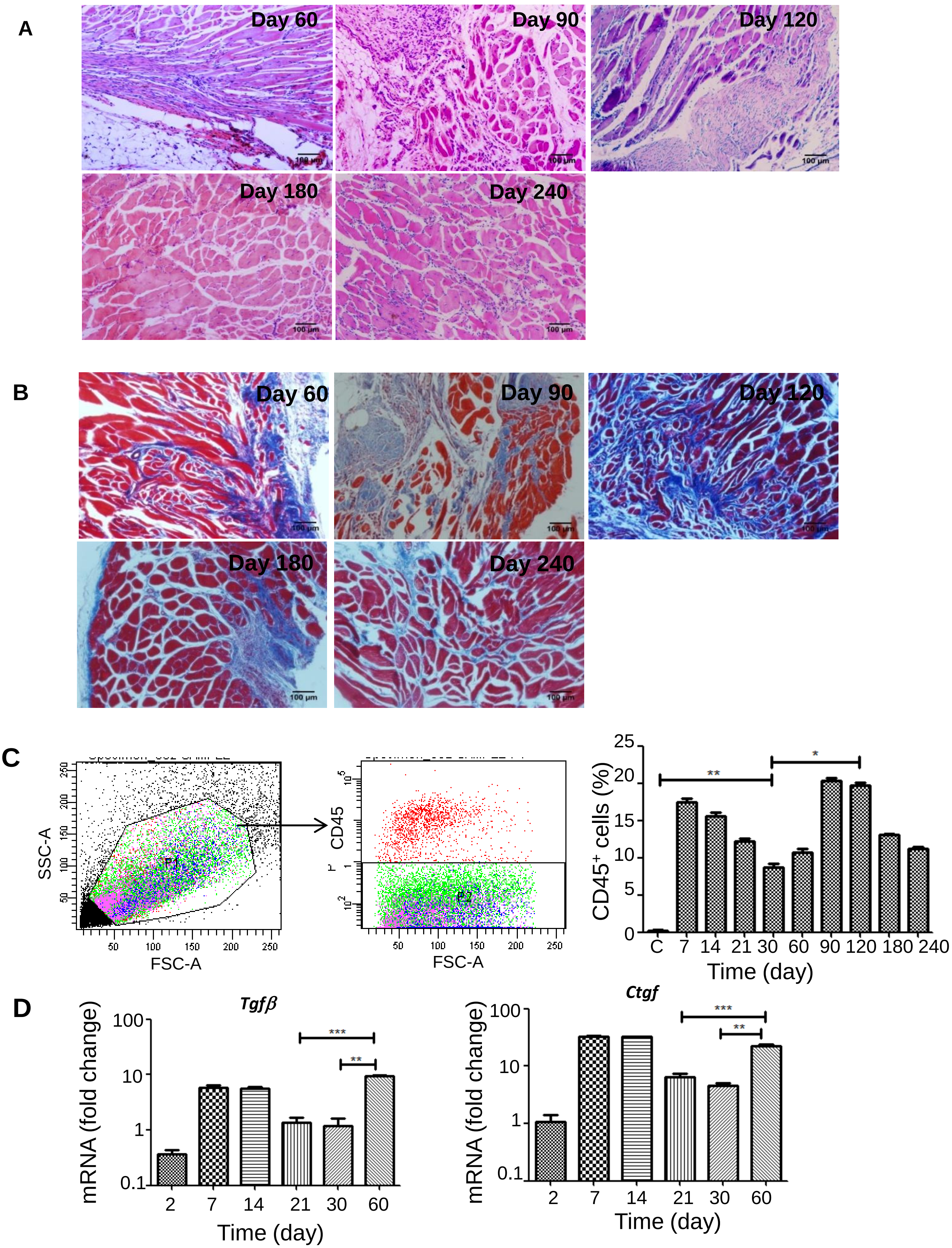

Supplementary Fig.2 (S2)

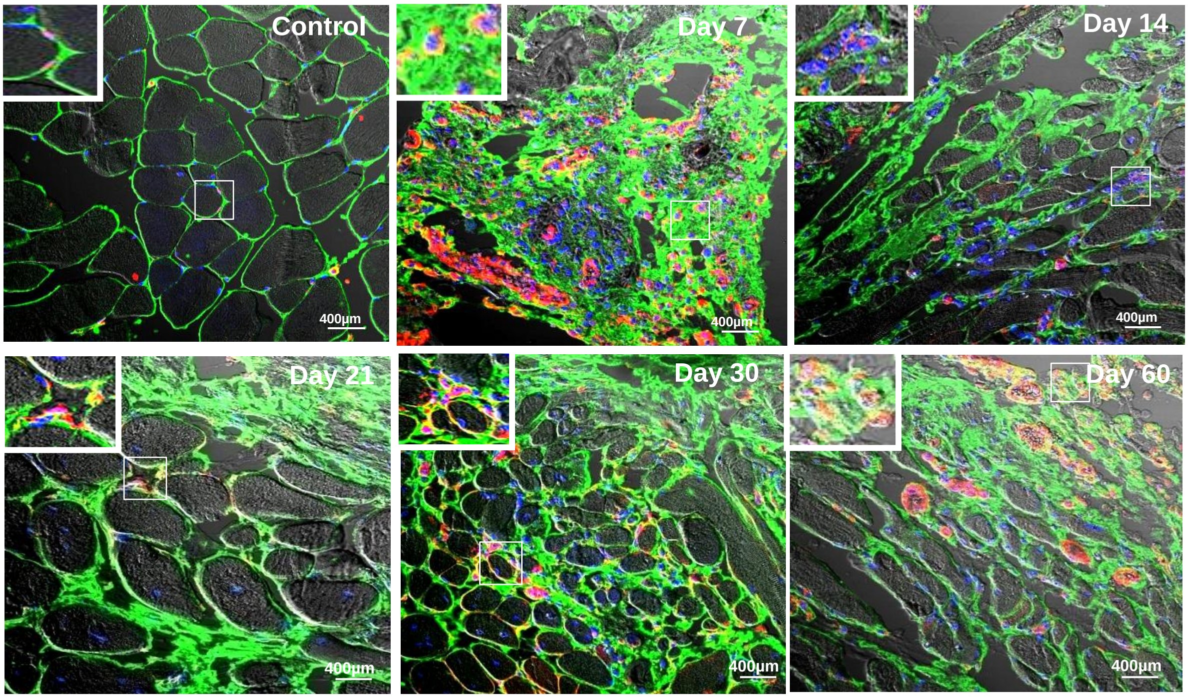

Supplementary Fig.3 (S3)

A

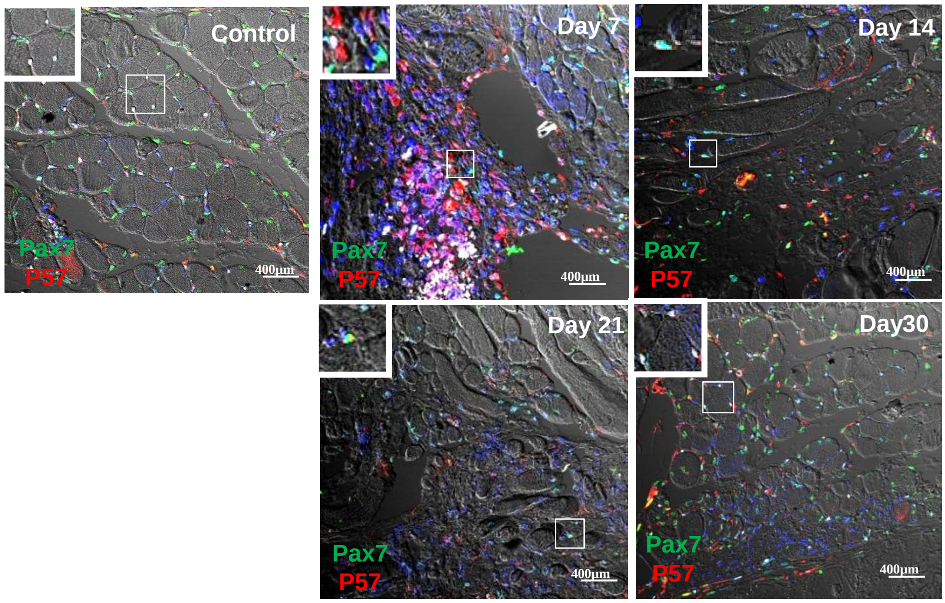

B

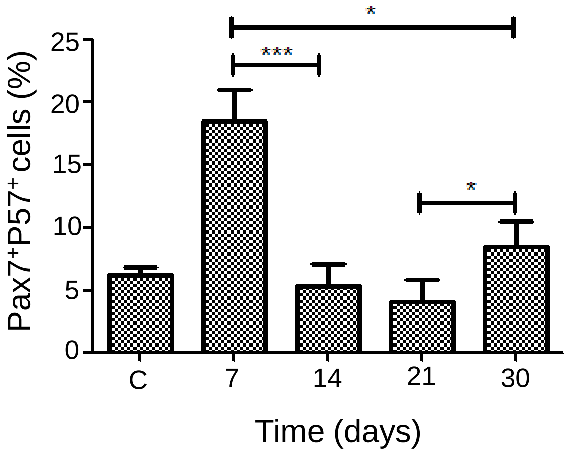

Supplementary Fig.4 (S4)

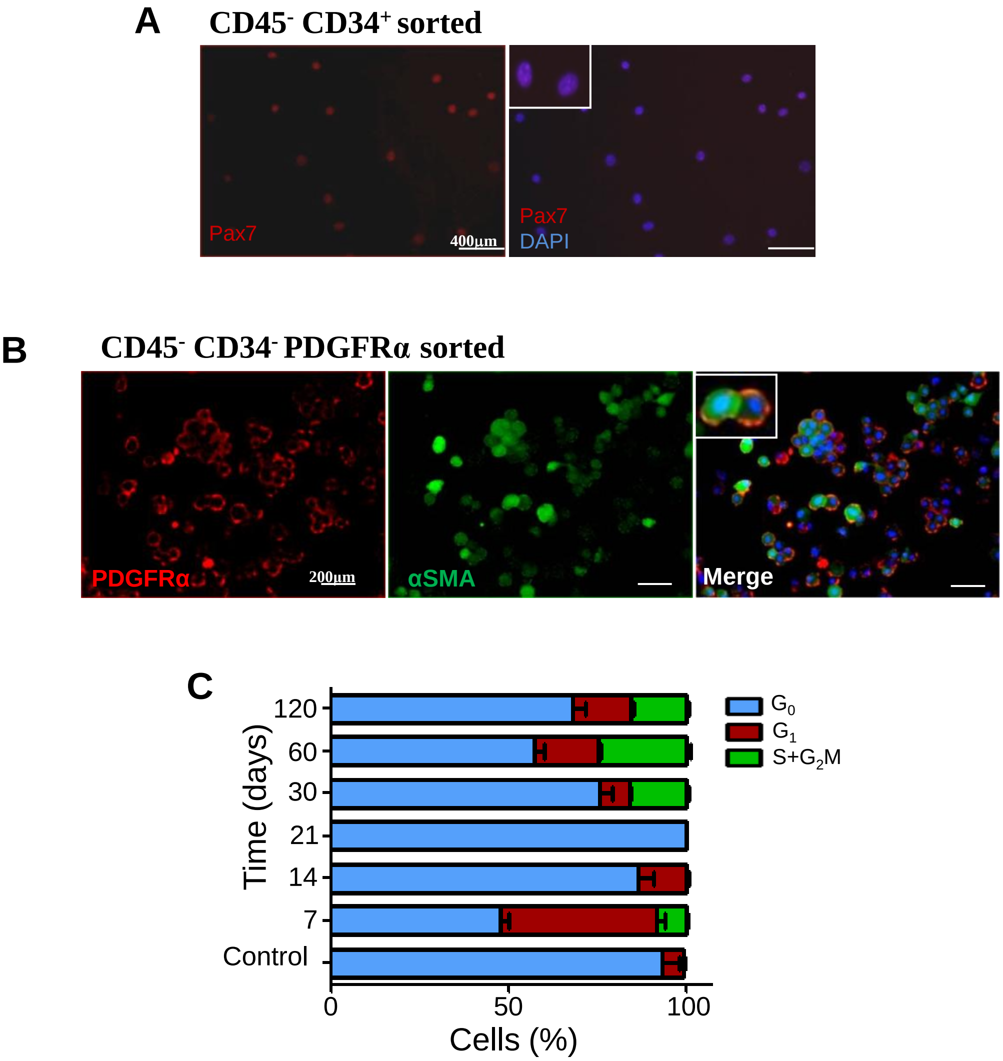

Supplementary Figure Legends

Figure S1. Aberrant regeneration and inflammatory response. A & B. Histopathological evaluation of tissue sections. H&E and Masson's trichrome stain of tissue sections post days 60, 90, 120, 180 and 240 of inflicting injury. C. Representative dot-plots show the presence of infiltrated leucocytes ($CD45^+$) in the chronically injured muscle post 60 days. The bar diagrams represent the time course of the leucocytes ($CD45^+$) infiltration. D. Relative gene expression in the injured skeletal muscle tissue. Whole cell gene expression for *Tgfβ* and *Ctgf* are shown. Relative fold change was calculated with respect to fibroblasts present in normal skeletal muscle tissue. Data were presented as mean ± SEM. ***$p<0.001$, **$p<0.01$,* $p < 0.05$.

Figure S2. Immunohistological analysis of tissue fibrosis. Tissue sections were co-stained with collagen Type I and αSMA antibodies at different time intervals of the injury.

Figure S3. Quantitative analysis of the quiescent SCs. A. Confocal images show the presence of $Pax7^+P57^+$ cells over the time course of the recovery. B. Bar diagrams show morphometric analysis of the quiescent SCs ($Pax7^+P57^+$). Analysis was based on 21 fields with 40x magnification at each time points. Data were presented as mean ± SEM. *** $p < 0.001$, * $p < 0.05$.

Figure S4. Immunocytochemical analyses of SCs and MFs. A. Analysis of sorted SCs. The expression of Pax7 is shown in $CD45^-CD34^+$ cell population. B. Analysis of MFs. The expression of PDGFRα is shown in $CD45^-CD34^-$ cell population. C. Cell cycle analysis of MFs. $CD45^-CD34^-PDGFR\alpha^+$ cells are subjected to cell cycle analysis by labeling with Hoechst 333342 and Pyronin Y dyes.

Supplementary Methods

Paraffin block preparation

Injured skeletal muscle tissue was fixed in 1X zinc fixative for 72 h. The tissue was washed with distilled water for 15 min (change of water in every 5 min). The samples were dehydrated by placing the fixed tissues in increasing grades of isopropanol (50%, 70%, 90% and 100%) for 1h incubation at 40°C. Tissues were then immersed in a mixture of xylene and isopropanol (1:1) for 30 min, followed with immersion in Xylene for 30 min with 3 changes, all at 40°C. The tissues were then placed in a mixture of xylene and Paraffin wax at 60°C for 30 min. The tissues were shifted in to paraffin wax at 60°C for overnight, and then they were shifted for next two changes of paraffin for 1 h each. On completion of this process the tissues were embedded in paraffin wax in a suitable mold and 5 µm sections were cut using a microtome (MRS3500, Histo-Line Laboratories, Milan, Italy) for further analysis. The sections were deparaffinized in the reverse order of paraffinization by immersing the slides in the series of reagents. To begin with, the slides were kept for 10 min each in Xylene (3 changes), 100% (2 changes), 90%, 70%, 50% isopropanol. Slides were finally rinsed in double distilled water. The deparaffinized slides were stained with H&E for tissue histopathology and used for immunohistochemistry post antigen retrieval.

TUNEL Assay

The deparaffinized sections were washed by immersing in 0.85% NaCl for 5 min followed by immersion in PBS for 5 min. The sections were again fixed by immersing slides in 4% paraformaldehyde in PBS for 15 min and then 5 min PBS washes (twice). The sections were permeabilized by addition of 100 µl of 20 µg/ml Proteinase K solution and incubated at room temperature (RT) for 10 – 30 min followed by 5 min PBS wash. The sections were re-fixed in 4% paraformaldehyde in PBS for 5 min followed by 5 min PBS wash (2×). The sections were equilibrated by addition of 100 µl equilibration buffer provided in the DeadEnd™ fluorometric TUNEL system kit (Fischer Scientific, Cat # G3250) at RT for 5-10 min. The equilibrated sections were labeled using 100 µl of TdT reaction mix. The sections containing the TdT reaction mix was covered by a plastic cover-slip to ensure even distribution of the mix. Incubate the slides for 60 min at 37°C in a humidified chamber. The reaction was stopped by removal of cover-slip and immersing the slides in 2X SSC buffer for 15 min. The sections were washed thrice in PBS (5 min). The sections were counter stained using DAPI for 10 min at RT and then mounted using ProLongR anti-fade reagent (Molecular probes) and visualized with Olympus BX51 fluorescence microscope. The digital images were acquired using DP70 camera (Tokyo, Japan) and processed with Image Pro software (Media Cybernetics, Inc., Rockville, MD).

Real Time PCR

Real-time qPCRs were performed by means of SYBR Green technology (Applied Biosystems, Foster City, CA) in Real time PCR instrument, Stratagene Mx 3000p (Agilent Technologies, Santa Clara, California). The total RNA obtained using TRIZOL Reagent (Invitrogen, Carlsbad, CA, USA) was treated with DNAse to facilitate the removal of genomic DNA. cDNA synthesized with the High capacity cDNA Reverse transcription kit from Applied Biosystems was analyzed for the expression of desired gene. Expression of gene of interest in samples was calculated as relative fold change using the formula $2^{-\Delta\Delta Ct}$. Sequences of primers are given in the Supplemental Table 2.

Western blot analysis

Injured tissue samples were collected from the mice at different time intervals. The cytoplasmic and nuclear extracts were normalized using SDS sample buffer (5% SDS, Tris-HCL: 189 mM, 30% glycerol and 15% β-Mercaptoethanol)[1]. The samples were resolved on 12% SDS-PAGE run for 20 min, initially at 80 V then 120 V. The SDS-PAGE gel and blotting pads (Biorad) was equilibrated using transfer buffer (192 mM Glycine, 25 mM Tris, 10% Methanol) in the transfer apparatus (Biorad). The PVDF (GE healthcare) was activated with water equilibrated with transfer buffer. Transfer was done at 12V (5 min) followed by 18V for 60 min. The membrane was washed with TBST (150 mM NaCl, 25 mM Tris and 0.1% Tween 20, pH 7.5) post transfer, followed by blocking with 5% skimmed milk (HI-Media) in TBST (60 min) at RT. The membrane is then incubated with primary antibody (in 3% skimmed milk in TBST) at 4^0C overnight on a rocker. The blot was washed extensively with TBST and then incubated with HRP conjugated secondary antibody (in 5% skimmed milk, TBST) at room temperature for 60 min. The blot was developed using ECL plus reagent (Pierce) and imaged with Typhoon 9400 Variable mode Imager (GE Healthcare). The blots were used to detect β-actin, and Phospho NF-κB proteins.

1. EI, D. MyoD and Myogenin protein expression in skeletal muscles of senile rats. *Cell Tissue Res.* **311**, 401-16, (2003).

Supplementary Table S1: Antibodies used in flowcytometry and immunohistological analyses.

**Name**	**Source**	**Conjugation**	**Dilution**	**Company**
CD34	Mouse	Biotinylated	1:100	eBiosciences (Cat # 13-0341)
CD45	Mouse	Biotinylated/APC	1:200	eBiosciences (Cat# 13-0451)
Pax7	Goat	Biotinylated	1:50	Novus Biological (Cat# NBP1 – 52374)
P57 – KP39	Mouse	Purified	1:50	Novus Biological (Cat# NB120-3223)
Pdgfra (CD140a)	Rat	Purified	1:100	BD Pharmingen (Cat# 558774)
Pdgfra (CD140a)	Rat	Monoclonal	1:100	Abcam (ab90967)
Myogenin	Mouse	Alexaflour 488	1:50	eBiosciences (Cat# 53-5643-80)
αSMA	Mouse	Purified	1:100	IMGENEX (Cat# IMG-80014)
Collagen Type1	Rabbit	Purified	1:100	Novus Biological (Cat# NBP1–30054)
Caspase 3	Mouse	Purified	1:100	Promega (Cat# G7481)
Foxo3a	Mouse	Purified	1:100	IMGENEX (Cat# Img6098A)
PhosphoNFκBP65 (Ser276)	Rabbit	Purified	1:50 / 1:200	Cell signaling Technology (Cat# 3037)
b-actin	Goat	Purified	1:200	Santa Cruz (Cat# SC1615)

Supplementary Table S2: Primers used in the RT-PCR analysis

**Gene**	**Forward Primer**	**Reverse Primer**	**Amplicon Size (bp)**
Tgf-β	*TTTGACGTCACTGGAGTTGT*	*GCTGATCCCGTTGATTTCCA*	148
Ctgf	*CCTGGTCCAGACCACAGAGT*	*TTTTCCTCCAGGTCAGCTTC*	148
Tnfα	*CACCACGCTCTTCTGTCTAC*	*AGAAGATGATCTGAGTGTGAGG*	144
Il1-β	*AACCTGCTGGTGTGTGACGTTC*	*CAGCACGAGGCTTTTTTGTTGT*	128
